# Functional Genetic Variation in *Ubiquitin D* Promoter Influences Susceptibility to Atopic Dermatitis

**DOI:** 10.1111/cea.70372

**Published:** 2026-06-14

**Authors:** Yi Ying Eliza Lim, Yang Yie Sio, Terence Yin Weng Lam, Yee‐How Say, Kavita Reginald, Fook Tim Chew

**Affiliations:** ^1^ Department of Biological Sciences, Faculty of Science National University of Singapore Singapore Singapore; ^2^ Department of Biomedical Science, Faculty of Science Universiti Tunku Abdul Rahman (UTAR) Kampar Campus Kampar Perak Malaysia; ^3^ Department of Biological Sciences, School of Medical and Life Sciences Sunway University Subang Jaya Malaysia

**Keywords:** atopic dermatitis, epistasis, I‐kappa‐B‐alpha (IκBα), nuclear factor kappa B (NF‐κB), single nucleotide polymorphisms (SNPs), ubiquitin D

## Abstract

- A ubiquitin D (UBD) promoter haplotype was identified to confer protection against atopic dermatitis (AD).
- The rs362513‐G allele suppresses UBD transcription and downstream NF‐κB–regulated cytokine production, independent of the AD‐risk SNP rs995185.

To the Editor,

1

Atopic dermatitis (AD) is a chronic inflammatory skin disorder driven by complex interactions between genetic susceptibility, immune dysregulation and epithelial barrier dysfunction. Central to AD pathogenesis is sustained activation of the nuclear factor kappa B (NF‐κB) pathway, which orchestrates the transcription of pro‐inflammatory cytokines and chemokines in both immune and epithelial compartments [1, 2]. While genome‐wide association studies (GWAS) have identified numerous AD risk loci, the majority reside in non‐coding regions and the functional mechanisms linking regulatory variation to disease remain poorly defined. Ubiquitin D (UBD, also known as FAT10) is a ubiquitin‐like modifier that promotes proteasomal degradation of phosphorylated IκBα, thereby facilitating NF‐κB nuclear translocation and transcriptional activity [3]. Elevated UBD expression has been reported in multiple inflammatory and immune‐mediated contexts, yet its genetic regulation and contribution to AD have not been previously elucidated.

We conducted a GWAS of 5323 Singaporean and Malaysian Chinese individuals, comprising 1261 ad cases and 4062 controls, using five genotyping arrays with imputation against the 1000 Genomes Phase III Beijing Han Chinese reference panel [4, 5]. A genome‐wide significant association was detected at chromosome 6p22.1 within the promoter region of *UBD*, led by rs995185 (*p* = 1.7 × 10^−15^; OR = 1.46, 95% confidence interval, CI = 1.33–1.61). This association was further validated in an independent Precision Health Research, Singapore (PRECISE‐SG100K) cohort comprising 741 ad cases and 14,123 controls of Singaporean Chinese individuals. Fixed‐effect meta‐analysis of both cohorts demonstrated that the minor allele ‘T’ of rs995185 was significantly associated with AD, with a pooled OR of 1.295 (95% CI = 1.206–1.390, *p* < 0.0001). Although a second promoter SNP of *UBD*, rs362513, did not independently reach genome‐wide significance and exhibited low linkage disequilibrium with rs995185 (*r* ^2^ = 0.13), its regulatory position prompted further haplotype‐level interrogation. In the GWAS cohort (*n* = 5323), haplotype analysis of these two variants revealed three common promoter haplotypes, among which the rs362513‐G/rs995185‐A haplotype (Haplotype 2) was significantly enriched in controls and conferred protection against AD (Figure 1A).

**FIGURE 1:**
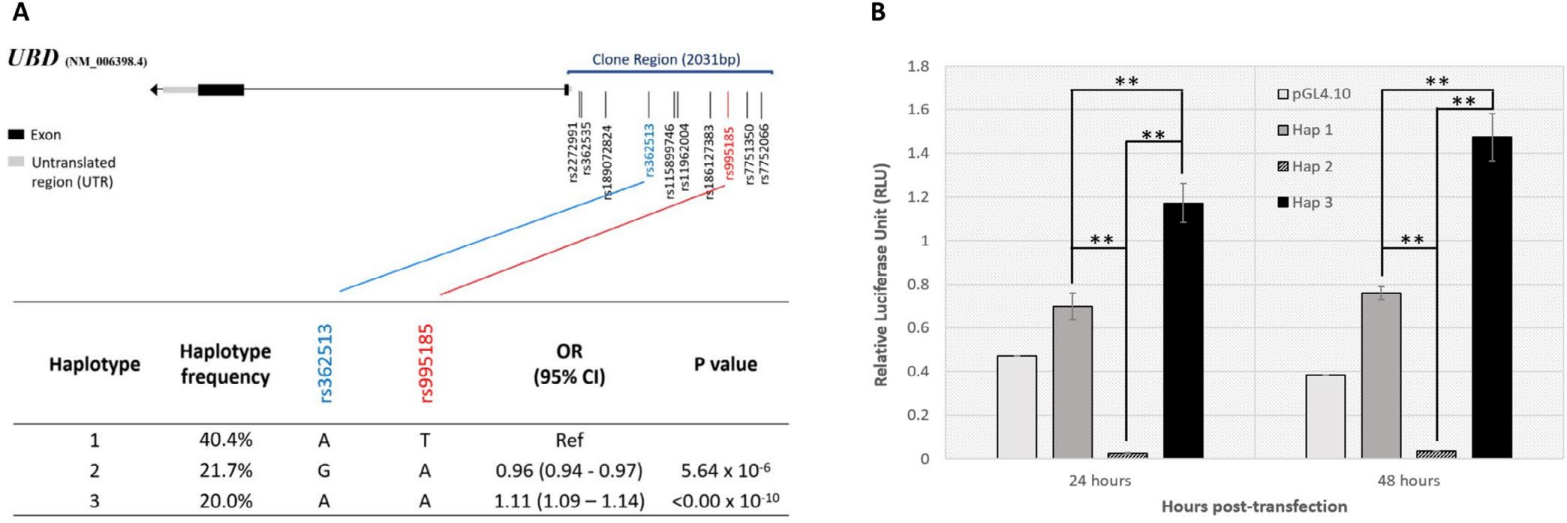
SNPs rs995185 and rs362513 association with AD and *UBD* promoter activity in the Singapore and Malaysia Chinese population. (A) *Ubiquitin D* (*UBD*) gene transcript variant NM_006398.4 gene structure with the relative positions of all 10 SNPs in *UBD* gene promoter region (2 kb upstream) found in the Singapore and Malaysia Chinese. AD‐associated SNP rs995185 (red font) and its linkage disequilibrium (*r* ^2^ > 0.13) SNP rs362513 (blue font) are shown respectively. Haplotype analysis shows haplotype 2 (21.7% frequency) to be associated with controls (*p* = 5.64 × 10^−6^). OR: Adjusted odds ratio, haplotype 1 as reference; CI: Confidence interval, lower 95% and upper 95% confidence interval; *p* value: Adjusted logistic regression *p*‐value. (B) Relative luciferase unit (RLU) of luciferase activity in HEK293T cells transfected with either *UBD* haplotype 1, 2, or 3. Haplotypes were cloned into the 5′UTR of pGL4.10 luciferase gene and measured 24‐ or 48‐h post‐transfection. Empty promoter‐less pGL4.10 included as background control. Data represent three independent experiments with triplicate measurements. ** indicates *t*‐test *p* < 0.01.

To characterize these promoter haplotypes, we cloned approximately 2 kb of the *UBD* promoter encompassing each haplotype into luciferase reporter constructs. In HEK293T cells, Haplotype 2 consistently exhibited markedly reduced promoter activity compared with the other haplotypes at both 24‐ and 48‐h time points (Figure 1B). Notably, this suppression occurred despite the presence of the rs995185‐A allele. These findings suggest a regulatory cascade in which rs362513‐G attenuates the effects of rs995185‐A, demonstrating how different promoter variants may modulate *UBD* expression.

The biological relevance of this regulatory architecture was supported by complementary ex vivo and in vitro data. In sera from genotyped individuals, Haplotype 2 carriers exhibited significantly reduced levels of NF‐κB‐regulated inflammatory mediators interleukin‐1α (IL‐1α) and programmed death‐ligand 1 (PD‐L1), consistent with attenuated NF‐κB signalling (Figure S1, Online Repository). To validate these effects, we expressed full‐length *UBD* cDNA under the control of each promoter haplotype in THP‐1 human monocytes. Cells expressing UBD driven by Haplotype 2 secreted significantly lower levels of canonical NF‐κB target cytokines IL‐1β and IL‐8 compared with cells expressing Haplotypes 1 and 3, reinforcing the functional impact of haplotype‐dependent UBD regulation on inflammatory output (Figure S2, Online Repository).

We further interrogated independent transcriptomic datasets derived from human skin biopsies to assess the disease relevance of UBD in AD (Figure S3, Online Repository). Across multiple cohorts, UBD mRNA levels were significantly elevated in lesional AD skin compared with non‐lesional skin and healthy controls [6, 7]. Importantly, *UBD* expression was consistently reduced following anti‐inflammatory interventions, including topical corticosteroid Betamethasone, calcineurin inhibitor Pimecrolimus and narrow‐band UVB phototherapy [8, 9], supporting a dynamic link between UBD and inflammatory disease activity.

Using HaCaT human keratinocytes, we demonstrated that UBD was significantly upregulated via NF‐κB activation following house dust mite (HDM) stimulation, a major environmental allergen that triggers AD inflammation (Figure S4, Online Repository). Importantly, UBD knockdown attenuated HDM‐induced IL‐1α upregulation, supporting its role in promoting NF‐κB‐associated inflammation in AD (Figure S5, Online Repository). Together, these data integrate genetic association, functional genomics and clinical transcriptomics to implicate UBD as a modulator of NF‐κB‐driven inflammation in AD.

While the associations described here were identified in a Singapore–Malaysia Chinese population and were not detected in large European or Japanese GWAS, this likely reflects population‐specific linkage disequilibrium structures and haplotype frequencies. Such differences underscore the importance of ancestry‐matched analyses and illustrate how regulatory variants with modest individual effects may act epistatically to shape disease risk. Consistent with this, rs362513 has not been prioritized in single‐variant analyses alone despite exerting substantial functional influence in a haplotype context.

To further strengthen and extend the present findings, ongoing and future studies will focus on expanding the sample size and validating these associations in additional cohorts. These efforts will enable finer resolution of haplotype effects, assessment of population generalizability and exploration of how these variants relate to clinically relevant disease features. Mechanistically, future studies will focus on defining allele‐specific transcription factor binding at the *UBD* promoter and elucidating how rs362513 variants may constrain NF‐κB signaling under inflammatory conditions.

In summary, we identify a promoter haplotype variant in *UBD* that gates NF‐κB‐associated inflammation, thereby modulating susceptibility to atopic dermatitis. These findings highlight the value of haplotype‐level functional dissection of non‐coding GWAS loci and identify a regulatory variant that influences inflammatory output in a disease‐relevant pathway.

## Author Contributions

F.T.C. conceived and supervised the overall research study. Y.Y.E.L. planned, conducted the experiments and analysed the data. Y.Y.E.L. wrote the manuscript. Y.Y.S. assisted in collecting and consolidating the data. T.Y.W.L. conducted experiments and analysed the data during manuscript revision. Y.‐H.S. and K.R. assisted in recruiting participants. All authors reviewed, revised and approved the final manuscript.

## Funding

Fook Tim Chew (F.T.C) received grants from the National University of Singapore (N‐154‐000‐038‐001 (E‐154‐00‐0017‐01); C141‐000‐077‐001 (E‐141‐00‐0096‐01)), Singapore Ministry of Education Academic Research Fund (R‐154‐000‐191‐112; R‐154‐000‐404‐112; R‐154‐000‐553‐112; R‐154‐000‐565‐112; R‐154‐000‐630‐112; R‐154‐000‐A08‐592; R‐154‐000‐A27‐597; R‐154‐000‐A91‐592; R‐154‐000‐A95‐592; R154‐000‐B99‐114), Biomedical Research Council (BMRC) (Singapore) (BMRC/01/1/21/18/077; BMRC/04/1/21/19/315; BMRC/APG2013/108), Singapore Immunology Network (SIgN‐06‐006; SIgN‐08‐020), National Medical Research Council (NMRC) (Singapore) (NMRC/1150/2008; OFIRG20nov‐0033; MOH‐001636 (OFLCG23may‐0038, A‐8002641‐00‐00)), National Research Foundation (NRF) (Singapore) (NRF‐MP‐2020‐0004), Singapore Food Agency (SFA) (SFS_RND_SUFP_001_04; W22W3D0006; NRF‐SFSRND2SIH‐0001; SFS_RND_2_FS_0002), Singapore's Economic Development Board (EDB) (A‐8002576‐00‐00) and the Agency for Science Technology and Research (A*STAR) (Singapore) (H17/01/a0/008; and APG2013/108). This research is supported by the National Research Foundation Singapore under its Open Fund‐Large Collaborative Grant (MOH‐001636) (A‐8002641‐00‐00) and administered by the Singapore Ministry of Health's National Medical Research Council. Kavita Reginald (K.R.) has received funding from the T20 Research Collaboration Grant Scheme from Sunway University with Grant No.: STR‐RMF‐T20‐005‐2019. The funding agencies had no role in the study design, data collection and analysis, decision to publish, or preparation of the manuscript.

## Ethics Statement

Study subjects from Singapore and Malaysia were recruited through ongoing cross‐sectional volunteer drives at the National University of Singapore (NUS), Sunway University and Universiti Tunku Abdul Rahman (UTAR). Ethical approvals were granted by the Institutional Review Board of NUS (References: NUS‐07‐023, NUS‐09‐256, NUS‐10‐445, NUS‐13‐075, NUS‐14‐150 and NUS‐18‐036), the Scientific and Ethics Review Committee of UTAR (Reference: U/SERC/03/2016) and the Sunway University Research Ethics Committee (Reference: SUREC 2019/029), in line with the Helsinki Declaration. Written informed consent was obtained on‐site via a Patient Information Sheet (PIS) for participants aged 18 and above and additional parental or guardian consent was required for those under 21. A subset of participants provided whole blood samples, collected by certified phlebotomists or nurses, with written informed consent and approval from the SERC of UTAR (Reference: U/SERC/03/2016), the Sunway University Research Ethics Committee (Reference: SUREC 2019/029) and the NUS IRB (References: NUS‐07‐023, NUS‐09‐256, NUS‐10‐445).

## Conflicts of Interest

F.T.C. reports grants from the National University of Singapore, Singapore Ministry of Education Academic Research Fund, Singapore Immunology Network, National Medical Research Council (NMRC) (Singapore), Biomedical Research Council (BMRC) (Singapore), National Research Foundation (NRF) (Singapore), Singapore Food Agency (SFA), Singapore's Economic Development Board (EDB) and the Agency for Science Technology and Research (A*STAR) (Singapore), during the conduct of the study; and consulting fees from Sime Darby Technology Centre; First Resources Ltd; Genting Plantation, Olam International, Musim Mas and Syngenta Crop Protection, outside the submitted work. The other authors declare no conflicts of interest.

## Data Availability

The datasets generated and analysed in this study can be obtained from the corresponding author upon reasonable request. Supporting Informations can be accessed through the repository at OSFstorage.
